# ChlamDB: a comparative genomics database of the phylum *Chlamydiae* and other members of the *Planctomycetes-Verrucomicrobiae*-*Chlamydiae* superphylum

**DOI:** 10.1093/nar/gkz924

**Published:** 2019-10-30

**Authors:** Trestan Pillonel, Florian Tagini, Claire Bertelli, Gilbert Greub

**Affiliations:** Institute of Microbiology, Lausanne University Hospital and University of Lausanne, Bugnon 48, 1011 Lausanne, Switzerland

## Abstract

ChlamDB is a comparative genomics database containing 277 genomes covering the entire *Chlamydiae* phylum as well as their closest relatives belonging to the *Planctomycetes-Verrucomicrobiae*-*Chlamydiae* (PVC) superphylum. Genomes can be compared, analyzed and retrieved using accessions numbers of the most widely used databases including COG, KEGG ortholog, KEGG pathway, KEGG module, Pfam and InterPro. Gene annotations from multiple databases including UniProt (curated and automated protein annotations), KEGG (annotation of pathways), COG (orthology), TCDB (transporters), STRING (protein–protein interactions) and InterPro (domains and signatures) can be accessed in a comprehensive overview page. Candidate effectors of the Type III secretion system (T3SS) were identified using four *in silico* methods. The identification of orthologs among all PVC genomes allows users to perform large-scale comparative analyses and to identify orthologs of any protein in all genomes integrated in the database. Phylogenetic relationships of PVC proteins and their closest homologs in RefSeq, comparison of transmembrane domains and Pfam domains, conservation of gene neighborhood and taxonomic profiles can be visualized using dynamically generated graphs, available for download. As a central resource for researchers working on chlamydia, chlamydia-related bacteria, verrucomicrobia and planctomyces, ChlamDB facilitates the access to comprehensive annotations, integrates multiple tools for comparative genomic analyses and is freely available at https://chlamdb.ch/. Database URL: https://chlamdb.ch/

## INTRODUCTION

All known members of the phylum *Chlamydiae* are obligate intracellular bacteria exhibiting a unique life cycle. Described chlamydial species cause a broad range of diseases in various species of birds, fishes, reptiles, amphibians, marsupials and mammals (1), and include major human pathogens such as *Chlamydia trachomatis*—a leading cause of blindness and infertility (1,2). *Chlamydiae* are difficult to cultivate and genetic manipulations are only available for a few species, which drastically slows down the understanding of their fascinating biology. Other members of the *Planctomycetes-Verrucomicrobiae*-*Chlamydiae* (PVC) superphylum include the closest relatives of the *Chlamydiae*: The *Planctomycetes* are extremely attractive for the field of evolutionary cell biology given their peculiar intracellular compartments (3). Like *Chlamydiae*, they replicate using an FtsZ-independent mechanism but contrarily to the *Chlamydiae*, *Planctomycetales* were shown to have a complete peptidoglycan cell wall (4–7). There is currently no database allowing an easy access and comparison of comprehensive genomics data for members of the PVC superphylum. A database focusing on the curation of chlamydial genome annotation was recently published (8), but it is limited to three species of the genus *Chlamydia*. A phylum-scale perspective including comparative data with the closest free-living relatives of the *Chlamydiae* would provide significant added value for the research community given the conserved intracellular lifestyle of these bacteria that were estimated to diverge over 700 million years ago (9). The PVCbase (10) provides updated automated protein annotations of forty-two PVC genomes, but only offers limited browsing capabilities and no comparative data. ChlamDB offers a centralized resource for genomic data and annotations of the entire PVC-superphylum. Its simple search engine allows browsing protein annotations, identifying orthologs in PVC genomes and performing a variety of comparative analyses.

### Genomic data and search

ChlamDB release 2.0 integrates data from 277 PVC genomes of 82 different species (Table 1), retrieved from GenBank (11) or RefSeq (12) (when GenBank records were not annotated). It includes all complete PVC genomes as well as draft genomes of the *Chlamydiae* phylum to increase the diversity of genera and species represented in the database. Draft genomes of the most studied *Chlamydia* species were discarded to reduce unnecessary redundancy in the database. Most genomes (*n* = 221) belong to the *Chlamydiae* phylum, including 86 *C. trachomatis*, 20 *Chlamydia muridarum*, 20 *Chlamydia psittaci* and 12 *Chlamydophilapneumoniae* genomes, thus allowing intra-species comparison for these important human pathogens. Species-level diversity was shown to determine *C. trachomatis* tissue tropism, hence showing the interest of such comparisons to elucidate novel aspects of chlamydial lifestyle and pathogenesis. To allow for broader comparisons, this database also contains the genomes of 34 *Verrucomicrobia*, 20 *Planctomycetes*, 1 *Lentisphaerae* and 1 *Kiritimatiellaeota*. Among the 34 *Verrucomicrobia*, there are 23 *Akkermansia muciniphila*, a bacterium commonly found in the human gut (13).

**Table 1.** Overview of ChlamDB content

Phylum	# genomes	# species
***Chlamydiae***	221	48
***Planctomycetes***	20	20
***Verrucomicrobia***	34	12
***Lentisphaerae***	1	1
***Kiritimatiellaeota***	1	1
**TOTAL**	**277**	**82**

The database provides various tools for comparing, analyzing and retrieving genomic data. A simple Boolean search interface allows querying the database for specific entries using NCBI protein accessions and locus tags or UniProt accessions. Accessions numbers of widely-used databases such as COG (14), KEGG ortholog (KO) (15), KEGG pathway (16), KEGG module, Pfam (17) and InterPro (18) are also recognized and can be used to search for proteins with specific annotations. The annotation of individual genomes can be browsed in tables of genes that are accessible directly from the front web page. In addition, sequence homology searches can be performed through a BLAST interface integrating the different blast flavours (BLASTp, BLASTn, tBLASTn and BLASTx) (19).

### Individual protein annotation view

**Figure 1.**
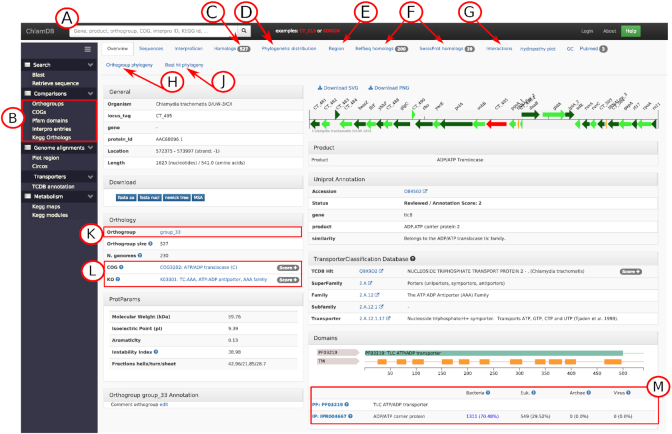
Protein annotation page of CT_495, an ADP/ATP transporter. (**A**) Main search bar. (**B**) Menu to access comparative analyses tools for the comparison of genome content based on the clustering of proteins into orthologous groups and for comparison of COG, KEGG, Pfam and InterPro annotations. (**C**) ‘Homologs’ tab with the list of the 527 orthologs of CT_495 in other PVC genomes. (**D**) Tab with the reference species phylogeny and the pattern of presence/absence of orthologs in each genome of the database as well as the locus tag of the closest ortholog in each genome. (**E**) ‘Region’ tab showing the conservation of proteins encoded in the direct neighborhood of the target protein. (**F**) Best hits in RefSeq and SwissProt databases. (**G**) Predicted protein interactors based on phylogenetic profiling and conservation of gene neighborhood. (**H**) Phylogenetic trees of the orthogroup and associated Pfam and transmembrane (TM) domain organization of each protein. (**J**) Phylogenetic tree including the best RefSeq hits of each protein of the orthologous group. (**K**) Name of the orthologous group with link to an overview of the annotation of the considered orthogroup (here 527 orthologs for group_33). (**L**) COG and KEGG annotations with link to the detailed list of proteins annotated with the same COG/KO in other genomes of the database; (**M**) Pfam and InterPro annotations with basic taxonomic information from the InterPro website: the numbers and percentages of proteins harboring this domain that are classified as Bacteria, Eukaryote, Archaea and Virus (data retrieved from InterPro version 60). Clicking on the Pfam accession numbers links to more detailed taxonomic information and a detailed list of proteins harboring the same domain in 6677 representative RefSeq genomes.

**Figure 2.**
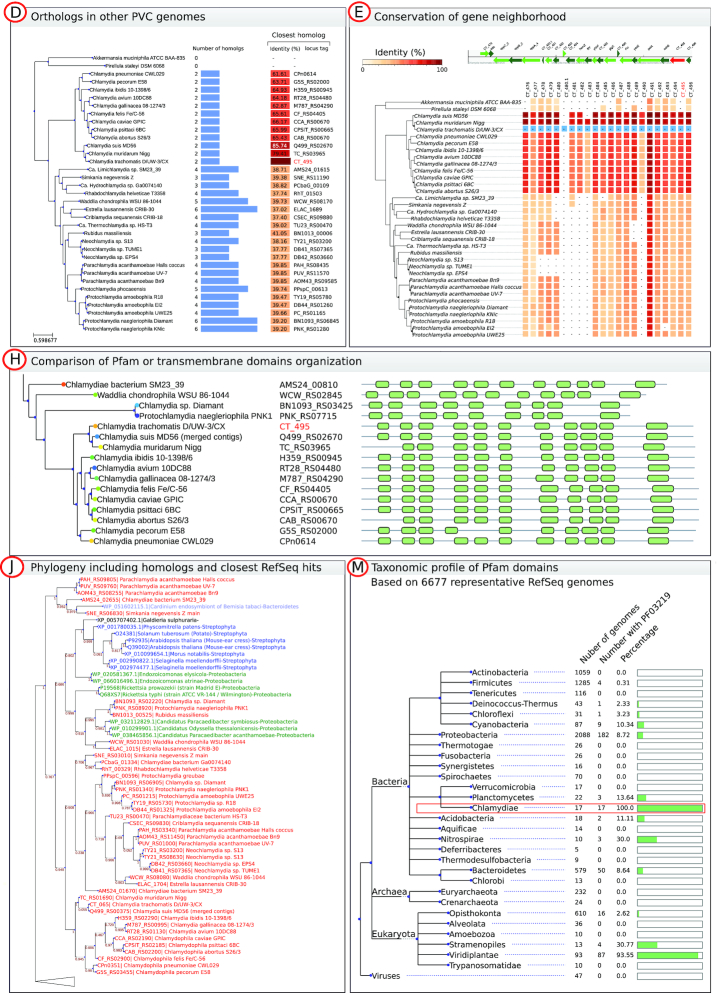
Selected examples of comparative data that can be retrieved from protein annotation pages. Panels are named according to the links shown in Figure 1. (**D**) Profile of presence/absence of orthologs in other PVC genomes with the identity of the closest ortholog in each genome. (**E**) Visualization of the conservation of proteins encoded in the neighborhood of CT_495. (**H**) Comparative view of transmembrane domains organization of CT_495 (TlcB) orthologs. (**J**) Phylogeny of CT_495 orthologous group including the closest identified RefSeq homologs. Red labels are proteins from the ChlamDB database whereas blue and green labels indicate non-PVC proteins. In this example, a sequence of *Cardinium*, a *Bacteroidetes* endosymbiont of the whitefly *Bemisia tabaci*, is clustering with *Chlamydiae* spp. and Proteobacteria symbionts such as *Paracaedibacter* and *Rickettsia* spp. are clustering with other *Chlamydia* spp., suggesting multiple events of horizontal gene transfer. Panels present only a subset of the 277 genomes currently present in the database to fit on a single page. Complete figures can be retrieved from the ChlamDB website. (**M**) Overview of the taxonomic profile of the Pfam domain PF03219 in 6677 representative RefSeq genomes. We can observe that this domain can be found in 100% of *Chlamydiae* genomes and in most *Viridiplantae*, but also in some genomes of other bacterial phyla. The detailed list of hits can also be browsed and downloaded.

Searching for a protein allows to access a ‘locus’ page, designed to summarize automated and imported functional annotations, and provides comprehensive comparative data to facilitate the interpretation of annotations (Figure 1). It integrates annotations from multiple databases including UniProt (curated and automated protein annotations) (20), KEGG (annotation of pathways), COG (orthology), TCDB (transporters) (21), STRING (protein-protein interactions) (22) and InterPro (domains and signatures). The different tabs at the top of the page link to additional data such as the list of orthologs in other PVC genomes (Figure 1C), identified using OrthoFinder (23). Orthologs are listed in a table containing the locus tag, the gene name, the name of the organism, the product, the percentage of amino acid identity as compared to the reference locus and the UniProt annotation score. Orthologs that were reviewed on SwissProt are flagged to quickly identify orthologs with manually curated annotations. Additional tabs link to (i) a precomputed phylogeny of the orthologous group, (ii) a second phylogeny that includes the closest non-PVC RefSeq hits of each sequence of the orthogroup, allowing to investigate the phylogenetic relationship of PVC proteins and their closest homologs available in public databases (Figures 1J and 2J), precomputed homology searches with (iii) RefSeq and (iv) SwissProt databases (200 top hits), (v) links to published literature based on text-mining from the STRING database (24) and PaperBLAST hits (25) and (vi) candidate functional interactors. Putative interactors were predicted in-house from genomic data alone using phylogenetic profiling and investigation of conserved gene neighborhood (see online methods) (Figure 1G). See (26) and (27) for the rationale justifying use of those two approaches.

We put a strong emphasis on the visual representation of the data (Figure 2). The pattern of presence/absence of orthologous groups within the PVC superphylum can be visualized with help of an annotated reference phylogeny (Figures 1D and 2D). The reference phylogeny was reconstructed with FastTree (28) (default parameters, JTT+CAT model) based on the concatenated alignment of 32 single copy orthologs conserved in at least 266 out of the 277 genomes.

The organization of transmembrane and Pfam domains in orthologs can be easily compared along the phylogeny of the orthologous group (Figures 1H and 2H). The conservation of proteins encoded in the direct neighborhood (23 kb upstream and downstream) of the protein of interest can also be visualized (Figures 1E and 2E).

The ‘orthogroup’ link (Figure 1K) provides an overview of the annotation of orthologs including gene name, product, COG annotation, KEGG annotation, InterPro annotations, number of transmembrane domains and sequence length. It allows verifying the consistency of annotations among putative orthologs and identifying wrongly grouped proteins (e.g. non-orthologous proteins sharing a domain).

### Annotation of candidate type III secretion system effectors

*Chlamydiae* use a type III secretion system (T3SS) to deliver effector proteins that will allow the bacterium to overcome eukaryotic host defenses and to manipulate host cells. Effectors are difficult to identify because they evolve quickly and are much less conserved than proteins encoding components of the T3SS apparatus (29,30). Between 5 and 8% of *Chlamydia* spp. coding sequences (CDS) are estimated to be effectors (31). Candidate T3SS effectors were identified using four different machine-learning classifiers that were trained with known effector sequences: BPBAac (32), effectiveT3 (33), DeepT3 (34) and T3_MM (35). In addition, we tagged proteins harboring eukaryotic domains rarely found in bacterial genomes. Such domains are known to be frequently involved in bacteria–host interactions (36,37). The ADP/ATP transporter domain (InterPro accession IPR004667) is for instance frequently found in both bacteria (70.48%) and eukaryotes (29.52%) (Figure 1L). A dedicated page allows visualizing the taxonomic distribution of each COG and Pfam domains across respectively 2,031 (for COG) and 6,677 (for Pfam) representative Archaea, Bacteria, Eukaryotes and Viruses genomes (Figure 1M and 2M). The detailed list of identified homologs can (for instance) be used to quickly determine whether a candidate effector protein harbors a domain predominantly identified in the genome of eukaryotes and other intracellular bacterial parasites such as *Rickettsia* or *Legionella*.

### Comparative genomics and data mining tools

Since *C. trachomatis* genome became one of the first sequenced genomes (38), hundreds of *Chlamydiae* genomes have been sequenced. Comparisons of complete genomes of different strains and species can help identify genetic variations that can be involved in defining tissue tropism or host specificity (39), or identify genes essential to the unique intracellular lifestyle of *Chlamydiae*. ChlamDB allows users to perform various comparative analyses based on orthologous proteins to identify highly conserved and genome-specific or clade-specific orthologous groups (Figure 3.1 and 3.2). Whole genome comparisons can be visualized using interactive circular genome maps, Venn diagrams or heat maps (Figure 3.3, 3.4 and 3.5). In addition, ChlamDB enables the alignment of local genomic regions in two or more genomes (Figure 3.6).

**Figure 3.**
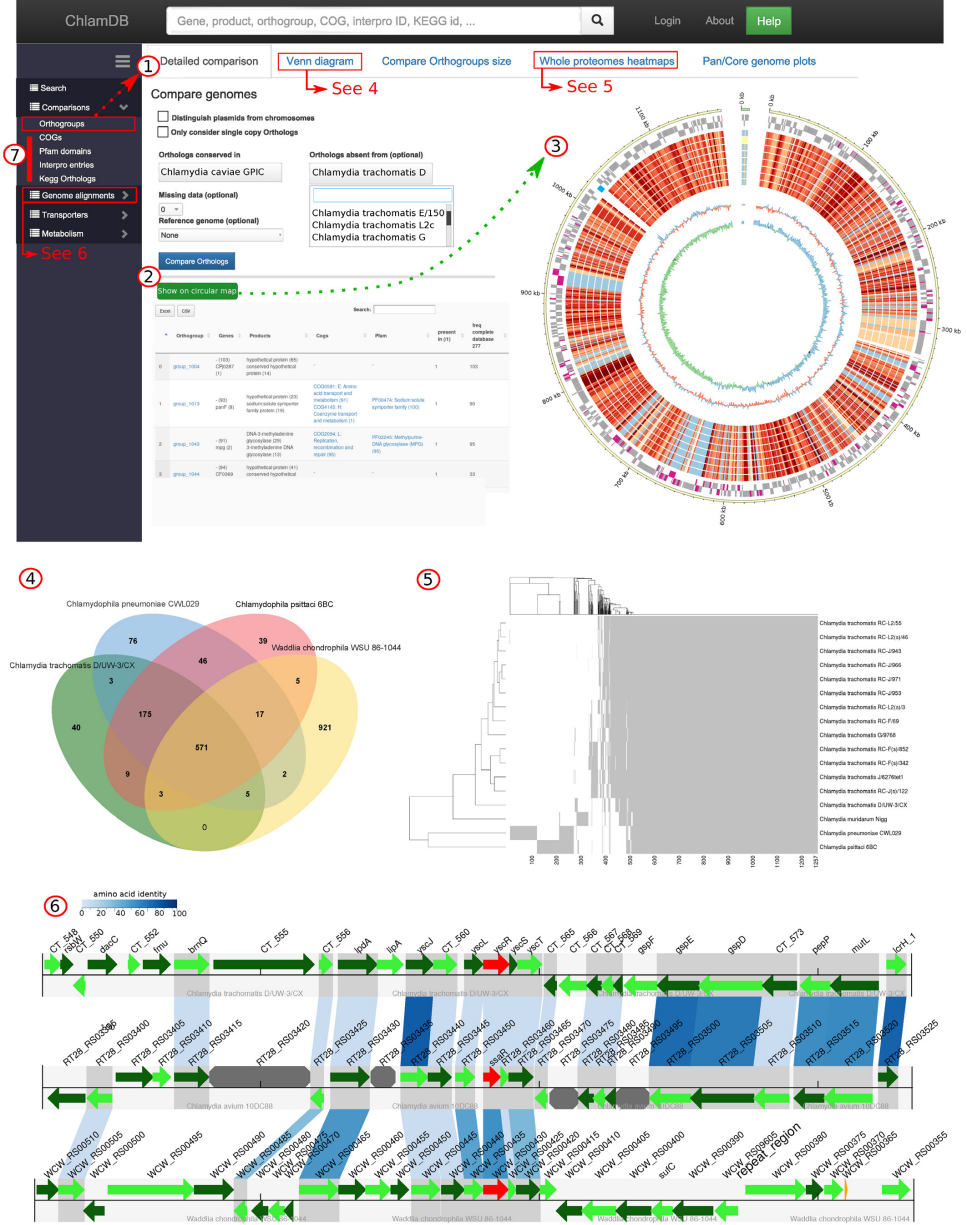
Comparative analyses based on orthologous groups. (**1**) The orthogroup comparison allows retrieving (**2**) a list of orthologous groups that are conserved in a group of genomes and absent from another group of genomes, that can be visualized on a circular map of one reference genome (**3**). The outer gray circles indicate CDS encoded on the leading and lagging strand of the genome. Proteins encoded in the genome of *Chlamydia caviae* and absent from the genomes of eight *Chlamydia trachomatis* strains are highlighted in pink. The inner red/blue circle indicates the conservation of each protein in the genomes included in the comparison (the red scale reflects protein identity, blue indicate absence of identified homolog). Regions of interest can be clicked, redirecting the user to the corresponding protein annotation page. (**4**) Up to six genomes can also be compared using interactive Venn diagrams. The complete list of shared or specific orthologous groups can be retrieved by clicking on the numbers. (**5**) Conservation of orthologous groups can be visualized as heat maps of the presence (gray) and absence (white) of each orthologous group in selected genomes. (**6**) Local alignments between distantly related genomes can be done using one locus (in red) as anchor. Green features are open reading frames (ORFs). Octagonal gray features are pseudogenes. Blue boxes linking ORFs from different genomes reflect the conservation of orthologs (the blue color scale reflects protein sequences identity). (**7**) Similar comparative analyses as in panels 1–5 can be done based on COG, Pfam, InterPro and KEGG orthologs annotations.

Pfam domains, KEGG orthologs and InterPro entries can also be compared to identify clade-specific or highly conserved protein features (Figure 3.7). A simple form enables the user to compare the size of gene families or the frequency of domains/KEGG annotations in each genome, allowing the identification of large protein families or frequent domains. For instance, the polymorphic membrane protein family (Pmp), a family of proteins involved in adhesion identified in all sequenced *Chlamydiaceae* genomes (40), is present in up to 28 copies in *C. psittaci* CP3 genome. Interestingly, the Pfam domain **PF05150** (‘*Legionella pneumophila* major outer membrane protein domain’), a domain extremely rarely identified outside of the *Legionella* genus (see https://chlamdb.ch/pfam_profile/PF05150/phylum) is present in 219 copies within the PVC superphylum (https://chlamdb.ch/fam/PF05150/pfam). This domain is also the most frequent domain identified in the genome of *Simkania negevensis* (36 occurrences). Proteins harboring this domain were probably acquired by horizontal gene transfer by *Chlamydiae*, *Legionella* or both and might share similar functions.

Annotations from the KEGG database were used to classify proteins into metabolic pathways and modules (16). Data for individual pathways and modules can be retrieved by searching KEGG accessions in the main search bar. In addition, KEGG annotations in various genomes can be compared as annotated phylogenies (Figure 4.1) and interactive bar charts or accessed from summary tables available for each genome (Figure 4.2). Modules and pathways pages detail KEGG orthologs associated to a given entry (Figure 4.3) and report the list of orthologs identified in each PVC genome (Figure 4.4).

**Figure 4.**
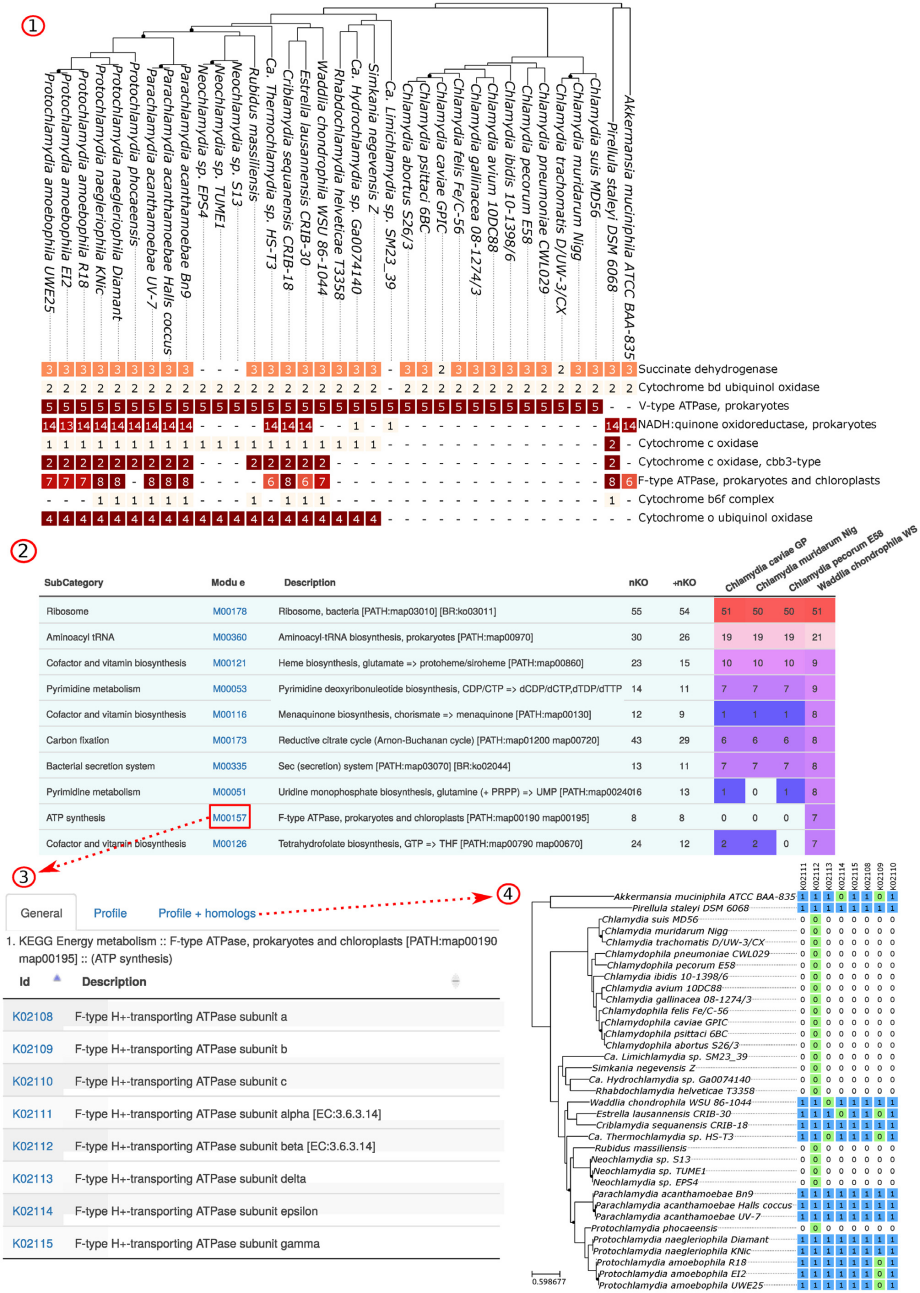
Comparative analysis of KEGG Pathways and Modules. (**1**) Comparison of KEGG modules of the ‘ATP synthesis’ category. Numbers indicate the amount of annotated Kegg Orthologs (KO) for each module. We can observe that F-type ATP-synthase subunits were identified in only some species of the phylum and were probably lost independently by *Neochlamydia*/*rubidus* strains and *Protochlamydia phocaensis*. (**2**) Module data can be browsed as tables. (**3**) Detailed lists of Kegg Orthologs (KO) can be retrieved from linked pages. (**4**) The pattern of presence or absence of each KO in each genome of the phylum can also be investigated visually (blue cells) for any module and pathway. Green cells indicate genomes for which no protein was annotated with the corresponding KO but an ortholog was identified based on OrthoFinder data. They could indicate either wrongly annotated proteins or non-orthologous proteins wrongly clustered in the same orthologous group. Panels 1 and 4 present only a subset of the 277 genomes currently present in the database to fit on a single page. Complete figures can be retrieved from the ChlamDB website.

### Implementation, methods and updates

The interface was developed using the Django framework (https://www.djangoproject.com/). Data are stored on a MySQL server and visualized with existing JavaScript libraries allowing to draw interactive plots and tables such as jvenn.js (41), datatables.js (https://datatables.net), cytoscape.js (42) and feature−viewer.js (https://github.com/calipho-sib/feature-viewer) (43). The python module GenomeDiagram is used to draw genome schematics, including alignments of multiple genomic locations (44). Circular representations of genomes and plasmids are made with Circos (45). The Ete3 Python module is used to draw phylogenetic trees with associated metadata (46). Some plots are also made using R (47), ggplot2 (48) and plotly (https://plot.ly). Annotations, phylogenetic trees and multiple sequence alignments can be downloaded from the website. A detailed description of the methods used to pre-compute functional and comparative analyses and setup the database is available online (https://www.chlamdb.ch/docs/index.html). The code source of the website is freely available on Github and issues can be reported online (https://github.com/metagenlab/chlamdb). This database has been developed at the Centre for Research on Intracellular Bacteria (CRIB) in Lausanne and will be maintained and updated at least once a year.

## CONCLUSION AND FUTURE DIRECTIONS

As the number of genome sequences quickly increases, there is a need for a centralized genomics resource providing updated annotations and extensive comparative genomics capabilities for the PVC superphylum. A superphylum-specific database has a significant added value with respect to large-scale genomic databases such as PATRIC (49) or Microscope (50): ChlamDB greatly facilitates access to comprehensive annotations and comparative data meaningful to the *Chlamydia* and *PVC* research community, with an intuitive interface and a special focus on visual representations of comparative data. Easy access to precomputed homology searches and phylogenetic reconstructions will help researchers to investigate the function and evolutionary history of proteins encoded in *PVC* genomes. Annotations of proteins specific for intracellular life such as predictions of type III secretion system effectors and identification of eukaryote-like domains will also facilitate the identification of uncharacterized proteins that might be involved in chlamydia-host interactions.

Since the annotation of PVC genomes stored in Genbank is generally not up-to-date with the most recent research, the existing ChlamDB could be extended to allow manual curation of the annotation and tracking of protein annotation history. Indeed, successful examples of community-curated databases exist for major pathogens, such as the Pseudomonas Database (www.pseudomonas.com) (51). The inference of orthologous relationships could be used to propagate the annotation of characterized proteins to less studied members of the phylum.
